# The evolution of drug-activated nuclear receptors: one ancestral gene diverged into two xenosensor genes in mammals

**DOI:** 10.1186/1478-1336-2-7

**Published:** 2004-10-12

**Authors:** Christoph Handschin, Sharon Blättler, Adrian Roth, Renate Looser, Mikael Oscarson, Michel R Kaufmann, Michael Podvinec, Carmela Gnerre, Urs A Meyer

**Affiliations:** 1 Division of Pharmacology/Neurobiology, Biozentrum of the University of Basel, Klingelbergstrasse 50-70, CH-4056 Basel, Switzerland; 2 (Present Address) Dana-Farber Cancer Institute, Harvard Medical School, Boston, MA 02115, USA; 3 (Present Address) Actelion Pharmaceuticals Ltd., CH-4123 Allschwil, Switzerland

## Abstract

**Background:**

Drugs and other xenobiotics alter gene expression of cytochromes P450 (CYP) by activating the pregnane X receptor (PXR) and constitutive androstane receptor (CAR) in mammals. In non-mammalian species, only one xenosensor gene has been found. Using chicken as a model organism, the aim of our study was to elucidate whether non-mammalian species only have one or two xenosensors like mammals.

**Results:**

To explore the evolutionary aspect of this divergence, we tried to identify additional xenobiotic sensing nuclear receptors in chicken using various experimental approaches. However, none of those revealed novel candidates. Ablation of chicken xenobiotic receptor (CXR) function by RNAi or dominant-negative alleles drastically reduced drug-induction in a chicken hepatoma cell line. Subsequently, we functionally and structurally characterized CXR and compared our results to PXR and CAR. Despite the high similarity in their amino acid sequence, PXR and CAR have very distinct modes of activation. Some aspects of CXR function, e.g. direct ligand activation and high promiscuity are very reminiscent of PXR. On the other hand, cellular localization studies revealed common characteristics of CXR and CAR in terms of cytoplasmic-nuclear distribution. Finally, CXR has unique properties regarding its regulation in comparison to PXR and CAR.

**Conclusion:**

Our finding thus strongly suggest that CXR constitutes an ancestral gene which has evolved into PXR and CAR in mammals. Future studies should elucidate the reason for this divergence in mammalian versus non-mammalian species.

## Background

A gene superfamily of heme-proteins, the cytochromes P450 (CYP), encodes the main enzymatic system for metabolism of structurally diverse lipophilic substrates [1]. A subset of these CYPs can be activated or inhibited in the liver by a variety of xenobiotic and endobiotic compounds. Transcriptional activation of these CYPs is part of an adaptive response to exposure to drugs and other xenobiotics and has major clinical and toxicological implications. The enzymatic capacities of the affected CYPs are changed, leading to an altered metabolic profile in the liver [2]. The barbiturate phenobarbital (PB) is prototypical for a class of compounds that induce or repress hepatic CYPs and many other genes [3]. PB-responsive enhancer units (PBRU) have been identified in the 5'-flanking regions of several of these CYPs and transcription factors binding to those units could be isolated (reviewed in [4-7]). In mammals, the pregnane X receptor (PXR, official nomenclature NR1I2) and the constitutive androstane receptor (CAR, NR1I3), both belonging to the gene superfamily of nuclear receptors, have been identified to be involved in hepatic drug-induction [8-12].

Strikingly, in contrast to the two xenobiotic-sensing nuclear receptors in mammals, only one xenosensor has been found in non-mammalian species, e.g. chicken [13], fish (fugu *Fugu rubripes *[14] and zebrafish *Danio rerio *[15]) or the nematode *Caenorhabditis elegans *[16]. The amino acid sequence of the full-length chicken xenobiotic receptor (CXR, NR1I3) is about equally related to those of mammalian PXRs and CARs [17]. Moreover, chicken CXR and mammalian PXR and CAR as well as drug-inducible CYP enhancer elements from these species could be freely interchanged in transactivation and electrophoretic mobility shift assays suggesting evolutionary conservation of the fundamental hepatic drug-induction mechanisms from birds to man [18].

In this report, we studied the evolutionary aspects of these findings. Despite using various methods and techniques, we were unable to isolate further genes that encode chicken xenobiotic-sensing nuclear receptors confirming the hypothesis that non-mammalian genomes only have one xenosensor gene. Since PXR and CAR exhibit different typical features concerning their activation, localization and regulation [6,19], we examined the properties of CXR to see whether on the functional and structural level, the chicken xenosensor shares common aspects with one or both of the mammalian receptors. Our findings give important insights the evolution of hepatic detoxification systems that protect different species from toxic compounds in their particular diet and environment.

## Results and Discussion

**Figure 1:**
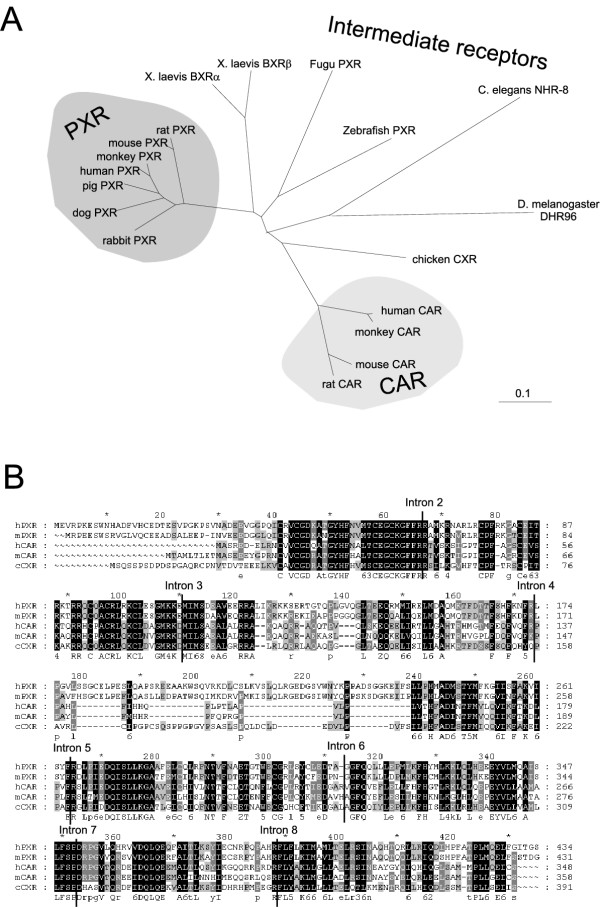
**Phylogeny of xenobiotic-sensing nuclear receptors from different species. ***A*, A non-rooted phylogenetic tree depicts the relationship between mammalian CARs and PXRs and non-mammalian intermediate receptors. The scale bar represents 0.1 amino acid substitutions per site. *B*, The sites of intron-exon junctions in the coding regions of CXR, PXR and CAR are highly conserved as depicted in an alignment of the amino acid sequences of these receptors.

Orthologs of PXR and CAR have been isolated from man, monkey, pig, dog, rabbit, mouse and rat [15]. In non-mammalian species, only one xenosensor gene is found and sequence-wise, the corresponding receptors from chicken, zebrafish, fugu fish and *C. elegans *are about equally related to the mammalian PXRs and CARs (Fig. 1A). Of the 18 nuclear receptors in the fruitfly *Drosophila melanogaster *genome, DHR96 shares considerable similarity to the xenosensors but the functions of this receptor have not been elucidated yet. Although the African clawed frog *Xenopus laevis *has two nuclear receptors, benzoate X receptor α and β (BXRα/β, NR1I2), that are related to the xenobiotic-sensing nuclear receptors, the BXRs are pharmacologically distinct from PXR and CAR and do not respond to xenobiotics [15,20]. No drug-sensing nuclear receptors have thus been isolated in amphibians so far. Figure 1A shows the phylogeny of the xenobiotic-sensing nuclear receptors from different species. The completion of the rat genome allowed a global analysis of the nuclear receptors from three mammalian species, man, mouse and rat. In the nuclear receptor subfamily NR1I which includes the 1,25-dihydroxyvitamin D_3 _receptor (VDR, NR1I1) in addition to PXR and CAR, intron-exon junctions are highly conserved [21]. Human and rodent CARs and PXRs have the same number of introns. Moreover, apart from one intron which is found in the variable region 5' of the DNA-binding domain, all other seven introns are located in the same position on the corresponding genes, even in the ligand-binding domains that in the case of CAR and PXR are unusually divergent for nuclear receptor orthologs [22]. Using a chicken genomic library, we isolated the gene encoding CXR and analyzed its structure. Again, the number of introns in the CXR coding sequence was the same as those in the mammalian xenosensors and the intron-exon junctions occur at the same locations (Figure 1B). The apparent conservation of gene structures between the single chicken xenosensors and the two mammalian orthologs suggest a close relationship between these receptors and supports the hypothesis that CXR constitutes an ancestral gene in chicken from which two receptors diverged in mammals.

To further test this hypothesis, we used different experimental approaches in order to isolate additional chicken xenobiotic-sensing nuclear receptors. Neither high- and low-stringency screening of a chicken liver cDNA library using CXR, CAR and PXR fragments as probes nor PCR-based strategies with degenerate primers designed on CAR and PXR alignments or degenerate primers based on generic nuclear receptor DNA-binding domains [23] resulted in the identification of novel chicken xenobiotic-sensing receptors (data not shown). The sequences of the previously unknown chicken orthologs for estrogen-related receptor γ (ERRγ, NR3B3) and a partial fragment of ear2 (NR2F6) that were found in these screens have been deposited (Genbank accession numbers AY702438 and AY702439, respectively).

**Figure 2:**
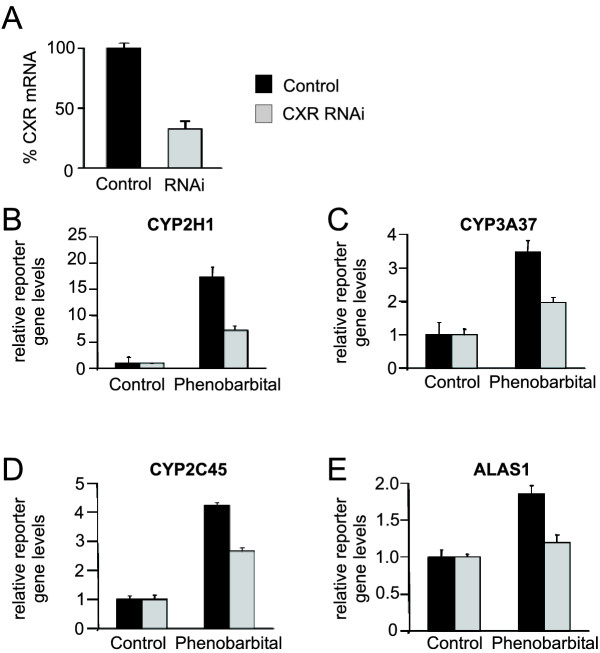
**Reduced drug-induction of drug-responsive enhancer elements from CYP2H1, CYP3A37, CYP2C45 and ALAS1 in LMH cells stably expressing CXR RNAi. ***A*, mRNA levels of endogenous CXR in LMH cells expressing pSUPER expression vector or CXR RNAi. CXR levels were measured by real-time PCR in LMH cells that stably express control vector or CXR RNAi. *B–E*, Phenobarbital-induction of drug-responsive enhancer elements from CYP2H1 (B), CYP3A37 (C), CYP2C45 (D) and ALAS1 (E) in LMH cells expressing pSUPER or CXR RNAi. LMH cells were transfected with the reporter gene plasmids and subsequently treated with vehicle or 400 μM PB for 16 hours before reporter gene levels were determined.

If CXR in fact is the only chicken xenobiotic-sensing nuclear receptor, ablation of CXR expression or function is predicted to drastically reduce drug-induction of CYPs and other target genes. To reduce CXR expression, we designed RNAi oligonucleotides targeting CXR and stably expressed those in the chicken hepatoma cell line leghorn male hepatoma (LMH). LMH cells express endogenous CXR and retain induction of genes by PB-type inducer compounds and other drugs [18]. As shown in Figure 2A, endogenous mRNA levels of CXR were reduced about 60% by the RNAi. LMH cells expressing either control vector or CXR RNAi were subsequently transfected with drug-responsive enhancer elements from CYP2H1 [17], CYP3A37 [24], CYP2C45 [25] and δ-aminolevulinate synthase (ALAS-1) [26] and treated with vehicle or 400 μM PB for 16 hours. ALAS-1 is the first and rate-limiting enzyme in heme biosynthesis and its transcription is regulated by a variety of factors and stimuli, including PB-type inducers and other drugs [26,27]. In the case of ALAS-1, the 2-fold PB-induction was completely abolished by the CXR RNAi (Figure 2E). In contrast, PB-activation of the CYP2H1, CYP3A37 and CYP2C45 PBRUs was only partially reduced by 50 to 60% (Figure 2B,2C,2D). In these cases, reduction of CXR levels by 60% might not be enough. Alternatively, these findings could also be explained by the presence of additional drug-sensing signalling mechanism independent of CXR.

**Figure 3:**
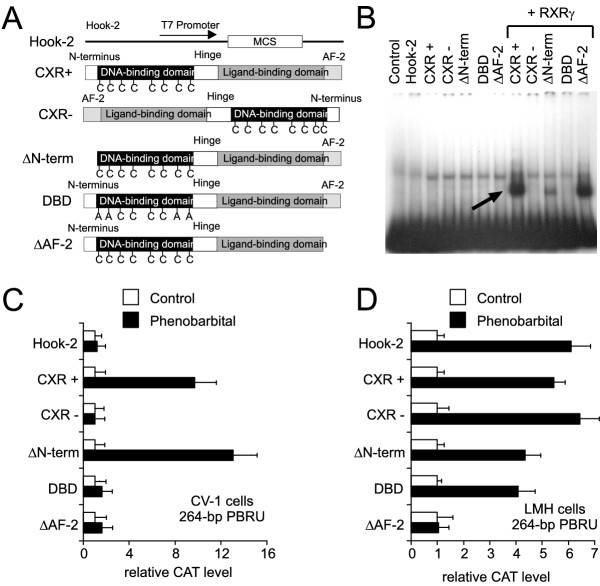
**Drug-induction of the 264-bp PBRU is abolished by a dominant-negative CXR allele. ***A*, CXR was subcloned into the pHook-2 expression plasmid (Hook-2) either full-length CXR in positive orientation (CXR+), negative orientation (CXR-), lacking its N-terminal amino acids 1–29 (ΔN-term), full-length CXR with four of its cysteine residues (cysteine 31, 34, 83 and 86) in the DNA-binding domain mutated (DBD) or lacking its C-terminal amino acids 383–391 containing the activation function AF-2 (ΔAF2). *B*, Electrophoretic mobility shift assays with mock *in vitro *transcribed/translated reticulocyte lysate (lane 1), expression plasmid pHook-2 (lane 2) and either expression plasmids for the different CXR alleles alone (lanes 3–7) or together with a pSG5-expression plasmid for chicken RXRγ (lanes 8–12). The arrow indicates the specific shift of CXR/RXR complexes with the radiolabeled CYP2H1 264-bp PBRU. *C*, pHook-2 expression plasmids without insert or containing the various CXR alleles were co-transfected with the CYP2H1 264-bp PBRU in the pBLCAT5 reporter vector as well as a lacZ-expression vector for normalization of transfection efficiencies into non-drug responsive CV-1 cells. After transfection, the cells were treated with either vehicle or 400 μM PB for 24 hours before cells were lysed and analysed for reporter gene expression and β-galactosidase expression. Values are the average of the relative CAT expression normalized for β-galactosidase levels of three independent experiments and error bars represent the standard deviation. *D*, pHook-2 expression plasmids without insert or containing the various CXR alleles were co-transfected with the CYP2H1 264-bp PBRU in the pBLCAT5 reporter vector into drug-responsive LMH cells expressing endogenous CXR. After transfection, the cells were treated with either vehicle or 400 μM PB for 24 hours before cells were lysed and analysed for reporter gene expression and β-galactosidase expression. Values are the average of the relative CAT expression normalized for β-galactosidase levels of three independent experiments and error bars represent the standard deviation.

Thus, we used an alternative method that aimed at reducing CXR activity by designing dominant-negative CXR alleles. These CXR mutants were then tested in reporter gene assays on drug-responding enhancer elements. In our case, we generated three different CXR alleles (Figure 3A): first, we deleted the N-terminus since in some nuclear receptors, this part harbours a ligand-independent activation domain AF-1 [28,29]. Second, site-directed mutagenesis of the cysteine residues in the zinc-fingers of the DNA-binding domain results in a CXR mutant that is expected to lack DNA-binding but to retain its ability to bind activators and to heterodimerize with its partner retinoid X receptor (RXR, NR2B1/2/3). Third, helix 12 in the ligand-binding domain was deleted which harbours a ligand-dependent activation domain AF-2. Nuclear receptors that act as dominant-negative alleles due to the absence of a functional AF-2 domain have been observed in some diseases (e.g. see refs. [30,31]). These findings were subsequently used to generate various dominant-negative nuclear receptor mutants for cellular assays [32].

First, the three CXR mutants were tested for their ability to bind to and activate a 264-bp PBRU isolated from the 5'-flanking region of chicken CYP2H1 [17,33]. As shown in electrophoretic mobility shift assays (Figure 3B), CXR can heterodimerize with RXR and bind to the 264-bp PBRU as wild-type, full-length receptor and when the N-terminal region from amino acid 1–29 (called ΔN-term) or the C-terminal region from amino acid 383–391 (referred to as ΔAF-2) are deleted, respectively (Figure 3B, lanes 8, 10 and 12). As expected, site-directed mutagenesis of four cysteine within the DNA-binding domain into alanine residues (denominated DBD) that participate in forming the zinc-finger domains abolishes protein-DNA interaction (lane 11). These results show that removal of the N-terminus or the C-terminus of CXR does not influence its binding to DNA. Subsequently, the CXR mutants were tested in CV-1 transactivation assays for functionality. The CV-1 monkey kidney cells constitute an excellent tool to study nuclear receptor function in a cellular system which does not express endogenous xenosensors, is not drug-inducible and thus has a very low background in these assays. Neither CXR lacking its C-terminal activation domain AF-2 (ΔAF-2) nor CXR with the mutated DNA-binding domain (DBD) are able to transactivate the CYP2H1 264-bp PBRU in CV-1 cell assays (Figure 3C). In contrast, removal of the N-terminus of CXR (ΔN-term) has no effect on its transactivation potential suggesting that no activation function AF-1 is present in these 29 amino acids. Finally, the test whether any of these CXR mutant alleles acts in a dominant-negative fashion is performed in the LMH cells which do express endogenous CXR and which are drug-inducible [18]. When co-transfected with the 264-bp PBRU, the CXR allele lacking a functional AF-2 domain (ΔAF-2) drastically decreases PB-induction of the PBRU (Figure 3D). In contrast, the DNA-binding domain (DBD) and the N-terminal truncated (ΔN-term) mutants have no effect. Similar results were obtained with PBRUs from other drug-responsive genes (data not shown). Together, the RNAi experiments and the findings using the dominant-negative CXR mutants show that functionally, CXR is the major drug-sensing nuclear receptor in chicken.

**Figure 4:**
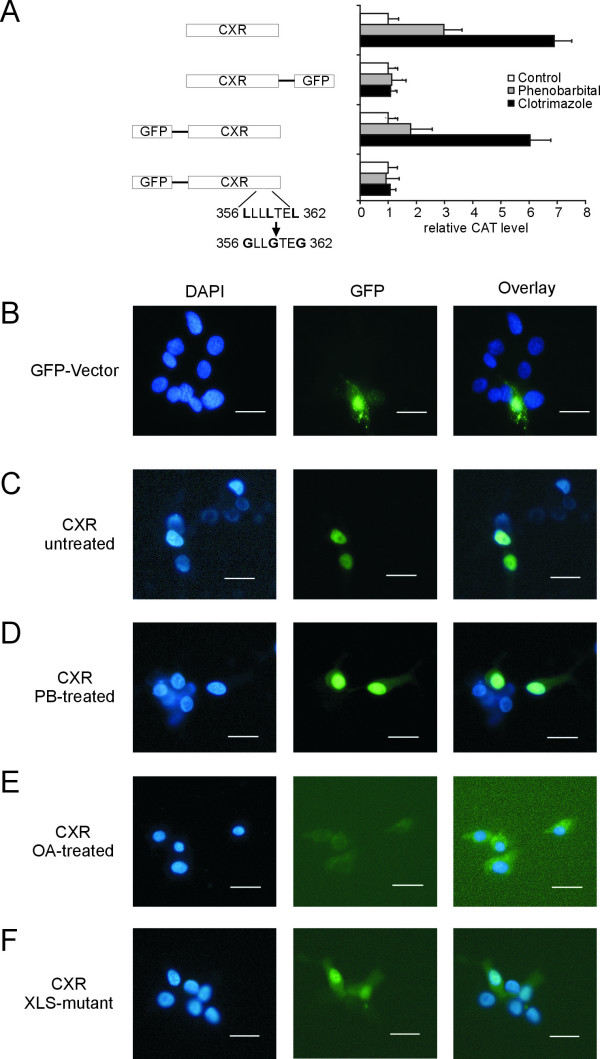
**Cellular localization of CXR in transiently transfected LMH cells. ***A*, Full-length CXR, CXR with GFP attached at its C-terminus or at its N-terminus or N-terminal GFP-CXR fusion protein mutated in its xenochemical response signal (XRS) at positions 356, 359 and 362 were expressed in CV-1 cells together with a reporter plasmid containing the CYP2H1 264-bp PBRU. After transfection, the cells were treated with either vehicle, 400 μM PB or 10 μM clotrimazole before cells were lysed and analysed for reporter gene expression. Values are the average of three independent experiments and error bars represent standard deviations. *B–F*, LMH cells were transfected with either pEGFP-vector alone (B), expression vector for N-terminal GFP-CXR fusion protein treated with vehicle (C), 400 μM PB (D) or 0.1 μM okadaic acid (E) for 16 hours or GFP-CXR fusion protein with the xenochemical response signal mutation as described in Fig. 4A (F). Cells were stained with 300 nM DAPI in PBS and analysed for DAPI and GFP-specific light emissions at 461 nm and 507 nm using excitation wavelengths of 358 nm and 488 nm, respectively. Size bars stand for 20 μm.

A significant difference between PXR and CAR in mammals is their mode of activation and their cellular localization [19]. PXR is strongly activated by a huge number of compounds. In contrast, CAR exhibits less promiscuity but high constitutive activity in most cellular assays [34]. However, CAR activity can be modulated by inverse agonists, agonists and different protein phosphorylation events [35]. In terms of activation, CXR is also highly promiscuous and normally has a low basal activity, thus pharmacologically more resembling a PXR-type than a CAR-type receptor [13,15]. Regulation of CAR activity can in part be explained by its unusual cellular localization. Both PXR and CAR undergo cytoplasmic-nuclear shuttling upon activation [36-40]. However, in contrast to PXR, CAR translocates after activation by PB, other xenobiotics or bilirubin for which no direct binding to the ligand-binding pocket was found. Although some progress in identifying CAR-interaction partners have been made recently [41,42], the mechanisms controlling the cytosolic-nuclear translocation are not clear. Interestingly, CAR translocation is independent of the C-terminal AF-2 function but instead requires the xenochemical response signal (XRS) LXXLXXL located between leucine 313 and leucine 319 in the human CAR sequence [37]. In contrast, cytoplasmic-nuclear translocation of VDR, the glucocorticoid receptor (GR, NR3C1) and the progesterone receptor (PR, NR3C3) is dependent on AF-2 suggesting a different mechanism for CAR shuttling [37,43]. A putative XRS (**L**LL**L**TE**L**) is also found in the CXR sequence between leucine 356 and leucine 362. Thus, to assess the relatedness of CXR to PXR and CAR in terms of cellular localization, we engineered different CXR-green fluorescent protein (GFP) fusion proteins. These were subsequently tested for functionality in CV-1 cell transactivation assays using the 264-bp PBRU as drug-sensitive enhancer. CXR with N-terminal, but not C-terminal GFP is activated by 400 μM PB and 10 μM clotrimazole after 16 hours of incubation (Figure 4A). Site-directed leucine to glycine mutagenesis in the CXR XRS reduces its ability to confer drug activation in these assays (Figure 4A).

**Figure 5:**
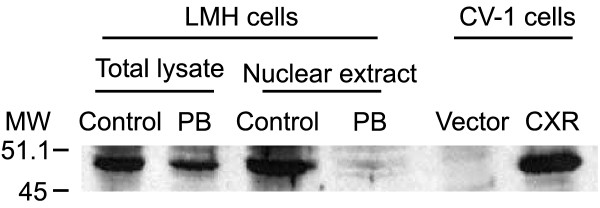
**Nuclear CXR protein levels decrease after PB-treatment. **LMH cells were treated with vehicle or 400 μM PB for 16 hours before cells were lysed and CXR protein levels in the total lysate (lanes 1 and 2) and in nuclear extracts (lanes 3 and 4) determined by Western blot. As controls, CV-1 cells were transfected with control vector or CXR expression vector (lanes 5 and 6). MW, molecular weight in kDa.

Subsequently, N-terminal GFP-CXR was transiently expressed in LMH cells, the cells were counterstained with DAPI to stain the nuclei and GFP-CXR localization was compared to that of GFP-expression vector without insert. GFP was found to be evenly distributed throughout the cell (Figure 4B). As depicted in Figure 4C, GFP-CXR in vehicle-treated LMH cells is exclusively in the nucleus. Treatment of transiently transfected LMH cells with 400 μM PB for 16 hours leads to an increase of GFP-staining in the cytosol (Figure 4D). Similar observations have been made for a variety of nuclear receptors where activation stimulates their export from the nucleus and subsequent degradation in the cytosol [44,45]. Accordingly, PB-treatment of LMH cells results in decreased CXR protein levels in total cell lysates (Figure 5, lanes 1 and 2) and even more dramatic in nuclear extracts (Figure 5, lanes 3 and 4) suggesting that activated CXR protein is more rapidly exported from the nucleus and degraded of this receptor. Most nuclear receptors that are exported and degraded upon activation share a conserved KXFF^K^/_R_R motif between the two zinc-fingers in the DNA-binding domain that can serve as binding site for calreticulin which is involved in the nuclear export [45]. PXR, CAR and CXR also contain a KGFFRR-motif but whether calreticulin plays a role in nuclear export of these receptors remains to be investigated. The protein phosphatase inhibitor okadaic acid inhibits PB-induction of mammalian and chicken PBRUs [13,18,46,47]. In transiently transfected LMH cells, 100 nM okadaic acid prevents nuclear localization of CXR after 16 hours (Figure 4E). Moreover, protein levels of the GFP-CXR fusion protein were reduced. Okadaic acid treatment prevents the drug-induced cytosolic-nuclear translocation of CAR [36]. Our findings regarding CXR are therefore very reminiscent of those results. Furthermore, site-directed mutagenesis of the XRS reduces the nuclear localization of CXR (Figure 4F) but not as completely as XRS mutations of CAR in mouse primary hepatocyte cultures [37]. The nuclear-cytoplasmic redistribution of this CXR mutant correlates with the decrease in its ability to activate the 264-bp PBRU in transactivation assays (Figure 4A). Thus, although CXR is normally found in the nucleus like PXR, it shares some features with CAR concerning its localization after treatment with okadaic acid or when its XRS is mutated.

**Figure 6:**
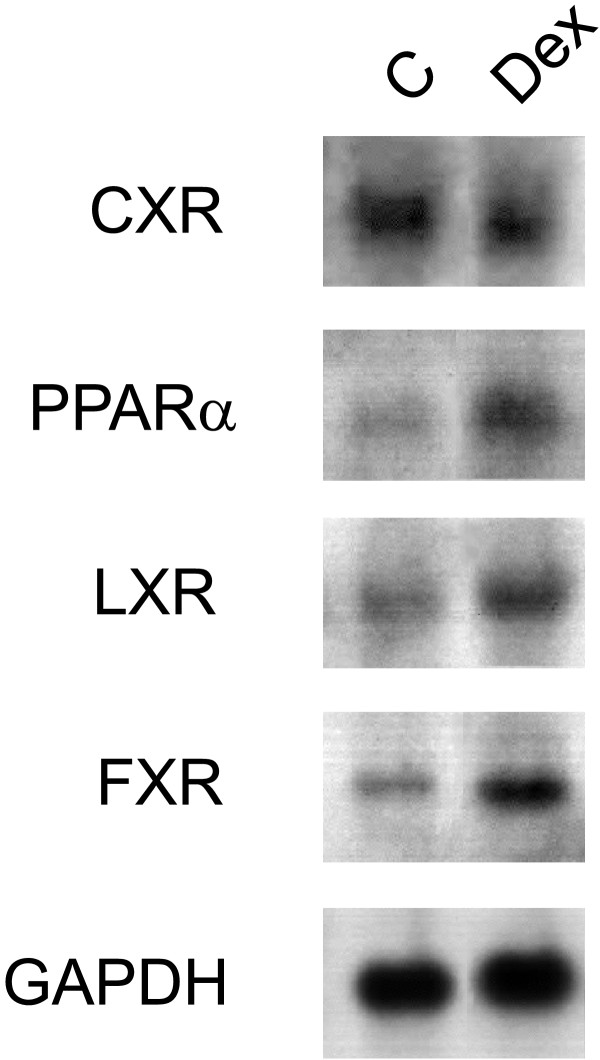
**Transcriptional regulation of chicken CXR, FXR, LXR and PPARα in LMH cells by glucocorticoids.  **LMH cells were treated for 16 hours with vehicle or 50 μM dexamethasone before cells were lysed and RNA was analysed by Northern blotting with probes for CXR, chicken PPARα, chicken LXR, chicken FXR or chicken glyceraldehyde 3-phosphate dehydrogenase (GAPDH).

In primary human hepatocytes, glucocorticoids have a dual effect on the expression of the drug-inducible CYP3A4 that is regulated by both PXR and CAR [48]. At low concentrations, these compounds activate GR which subsequently induces transcript levels of PXR and CAR [49,50] whereas higher concentrations of glucocorticoids directly activate PXR [9,51]. We thus wanted to test whether the chicken CXR is regulated in the same way as the mammalian xenobiotic-sensing receptors. Treatment of LMH with 50 μM dexamethasone (Dex) for 16 hours did not alter CXR expression (Figure 6). Moreover, dexamethasone does not activate CXR directly, at least at this concentration [13]. In contrast, dexamethasone increases transcription of the chicken peroxisome-proliferator activated receptor α (PPARα, NR1C1), the chicken liver X receptor (LXR, NR1H3) and the chicken farnesoid X receptor (FXR, NR1H4). These receptors play important roles in maintaining hepatic bile acid, cholesterol and lipid homeostasis, respectively [52]. PXR, CAR and CXR have been found to be activated by bile acids and thus are involved in the regulation of the intrahepatic levels of lipid soluble compounds by stimulating metabolism and subsequent excretion of these compounds [12,53,54]. Therefore, activation of one of these receptors leads to changes in intrahepatic lipid levels which then potentially affects transcription of the other receptors. However, the regulatory network of these receptors is still under investigation.

Molecular modelling studies confirm the close relationship between chicken and fish xenosenors to mammalian PXR. X-ray structures of human PXR revealed several peculiarities of the PXR ligand-binding domain which are not found in other nuclear receptors [55,56]. First, PXR has an expanded β-sheet with two more strands. Moreover, helix 6 is completely and helix 7 partially unwinded which leaves a solvent-accessible hole in the ligand-binding pocket that is capped by an extension in helix 1–3. Although an extended β-sheet is not obvious in chicken and fish xenosensors, both receptors have long helix 1–3 inserts which could potentially induce partial unwinding of helix 6 and 7. Thus, molecular modelling of aligned amino acid sequences suggest enlarged ligand binding pockets for both fish and chicken xenobiotic-sensing receptors which could explain their high promiscuity [15]. In striking contrast, CARs not only lack an extended β-sheet but also have a much shorter helix 1–3 resulting in a more rigid and less promiscuous ligand binding pocket [15,57,58]. Therefore, the relatively high degree of promiscuity of CAR could at least partially be due to the ability of different compounds to trigger cytoplasmic-nuclear translocation of this receptor independent of direct binding [35]. The loop connecting helix 11 and 12 is much shorter in the CAR sequence than most other nuclear receptors [15,57]. This short loop might reduce the ability of helix 12 and AF-2 to reach an inactive conformation and thus could explain the constitutive activity of CAR [57]. CAR also has a shorter helix 12 than most other nuclear receptors [57]. Interestingly, helix 12 of CXR is very conserved to that of mammalian CARs in terms of amino acid composition and of length whereas the length of the zebrafish xenosensor helix 12 is intermediate between CARs and PXRs.

## Conclusions

In summary, our results confirm that in contrast to mammals which have two xenobiotic-sensing receptor PXR and CAR, the genome of other species encodes for only one xenosensor. This hypothesis is supported by analysis in the fugu fish genome (data not shown), unsuccessful attempts to isolate further xenosenors in chicken and functional assays showing that ablation of CXR function drastically reduces drug-inducibility in a chicken hepatoma cell line. Our findings presented here and those of other laboratories imply that PXR and CAR origin from one ancestral gene which diverged into two genes in mammals. This ancestral gene, in chicken coding for CXR, is a promiscuous, PXR-like receptor. Thus, CXR and related receptors from fish are activated by a variety of different compounds [13,15]. Interestingly, in a comprehensive study of different classes of ligands on xenosensors from man, monkey, pig, dog, mouse, chicken and fish, CXR was one of the most promiscuous receptors in regard to the compounds tested [15]. Therefore, the ancestral xenosensors in non-mammalian species might have a broader substrate spectrum than their mammalian counterparts where the task for detoxification is split between two receptors [59]. On the other hand, CXR also shares some features with CAR that are not found in PXR: its short helix 12, the xenochemical response signal and in part its cellular localization after okadaic acid treatment. Finally, in contrast to both PXR and CAR, CXR is not regulated by glucocorticoid treatment in the chicken LMH cells suggesting that this regulation was acquired only after birds and mammals diverged from a common ancestor.

Evolution of drug-metabolizing CYPs and xenobiotic-sensing nuclear receptors is influenced by diet and exposure to other environmental chemicals. Accordingly, drug-induction is very species specific. This is reflected in the unusually divergent ligand-binding domains of PXRs and CARs orthologs [22]. When comparing PXRs and CARs from human, mouse and rat, nonsynonymous nucleotide substitution rates are considerably higher in comparison to any other nuclear receptor [21] and reflect the different evolutionary adaptations of these species to their specific environment. It is thus extremely puzzling why in non-mammalian species, one xenosensor is sufficient whereas two xenobiotic-sensing nuclear receptors have evolved in mammals. Furthermore, it is unclear why in addition to the ligand-activated PXR, mammalian genomes encode CAR, a nuclear receptor that is unorthodox in many ways. On one hand, CAR and PXR might just share the workload in hepatic detoxification of xenobiotics. On the other hand, evidence accumulated in recent years that both PXR and CAR have functions that go beyond detoxification. As example, PXR and CAR form an intricate network with other nuclear receptors and transcription factors to regulate hepatic cholesterol and bile acid homeostasis [60]. It is thus conceivable that these receptors have so-far unidentified functions in mammals which require two receptors and that are thus absent in non-mammalian species. Therefore, further insights into the evolution of drug-sensing nuclear receptors are extremely important in order to gain novel insights into the role of these factors in the physiology and pathophysiology of the liver.

## Methods

### LMH and CV-1 cell culture, transfection and reporter gene assays

Culture and transfection of LMH cells with FUGENE 6 Transfection Reagent (Roche Molecular Biochemicals, Rotkreuz, Switzerland) were performed as published [17,33]. Before transfections, LMH cells were kept in serum-free medium for 24 hours. CV-1 cell transactivation assays have been described in detail [17,33]. Sixteen or twenty-four hours after drug-treatment, cells were harvested and assays for CAT expression using a CAT ELISA Kit (Roche Molecular Biochemicals, Rotkreuz, Switzerland). CAT concentrations were normalized against β-galactosidase activities to compensate for different transfection efficiencies.

### Isolation of the CXR gene

Chicken BAC filters (UK Human Genome Mapping Project Resource Center, UK) were hybridised with a probe encoding for CXR. Positive clones were purchased, digested with different restriction enzymes and Southern blots obtained using the same probe. Bands hybridising with the CXR probe were isolated, subcloned and CXR genomic information obtained by PCR using primers designed after the CXR mRNA sequence.

### Site-directed mutagenesis

Mutagenesis was carried out using overlapping primers as described [17]. Mutated fragments were excised, cloned into new vectors and verified by sequencing.

### Electrophoretic mobility shift assays

Electrophoretic mobility shift assays have been described in detail [33]. Proteins were expressed using the TNT *in vitro *transcription/translation kit (Promega, Wallisellen, Switzerland) before being subjected to non-denaturing SDS-polyacrylamide gel electrophoresis with [^32^P]-radiolabeled CYP2H1 264-bp PBRU.

### Targeting of CXR in LMH cells by RNAi

Expression of CXR in LMH cells was repressed by RNAi as described [61]. In brief, a 19 bp fragment ranging from position 857 to 875 in the open reading frame of CXR was chosen for targeting. A double-stranded oligonucleotide containing this sequence and compatible ends for cloning into pSUPER was obtained by annealing single stranded oligonucleotides for the sense (GATCCCCGGATGGGGCTCTGGCCGGCTTCAAGAGAGCCGGCCAGAGCCCCATCCTTTTTGGAAA) and the anti-sense strand (AGCTTTTCCAAAAAGGATGGGGCTCTGGCCGGCTCTCTTGAAGCCGGCCAGAGCCCCATCCGGG) and subsequent ligation into pSUPER cut with BglII and HindIII (underlined letters refer to CXR-specific targeting sequence). After verification of the ligation product the pSUPER-CXR-RNAi expression cassette was cut out using BamHI and XhoI and subcloned into BglII/XhoI-digested pcDNA3 (Invitrogen, Carlsbad, USA). The ScaI-linearised construct was transfected into LMH cells using FUGENE 6 (Roche Molecular Biochemicals, Rotkreuz, Switzerland). Stable transfectants were selected by addition of 175 μg/ml G418 (PAA Laboratories, Pasching, Austria) to the cell culture medium. A control cell line was selected in parallel which was stably transfected with pcDNA3 carrying the empty pSUPER expression cassette. Reporter gene assays in LMH cells using the CXR-RNAi clones were performed using reporter constructs for CYP2H1, CYP3A37, CYP2C45 and ALAS-1 described previously [17,24-26].

### Cellular localization studies

LMH cells were cultivated on glass cover slips and subsequently transfected with pEGFP-C1 or pEGFP-N1 expression plasmids (Clontech, Allschwil, Switzerland) before cells were either treated with vehicle, 400 μM PB or 0.1 μM okadaic acid for 16 hours. Cells were washed with PBS, fixed in 3% formaldehyde for 30 minutes, washed again with PBS, stained with 300 nM DAPI and subsequently mounted on glass slides. Digital images were captured using a Leica DC 300F camera (Leica, Nidau, Switzerland) mounted on a Leitz DMRB microscope with the Leica IM50 Image Manager program version 1.20. Figures were assembled with Adobe Photoshop version 5.0.

### CXR antibodies, nuclear extracts and Western blots

CXR ligand-binding domain was expressed in bacteria, purified and injected into rabbits for antibody production according to standard procedures. Anti-CXR-ligand-binding domain antibody from rabbit serum was subsequently used in Western blots. LMH cells were grown under standard conditions and treated with vehicle or 400 μM PB overnight. Cells were subsequently washed with PBS and protein extracts prepared using RIPA buffer. As control, CV-1 cells were transfected with empty pSG5 expression vector or vector expressing CXR and subsequently lysed with RIPA buffer. Nuclear extracts were prepared as published [62].

### Northern hybridisation

LMH cells were treated with the indicated compounds for 16 hours before total RNA was isolated using the TRIZOL Reagent (Life Technologies, Basel, Switzerland). Twenty μg of total RNA were subjected to electrophoresis and analysed in Northern hybridisations as described [17,33].

## List of Abbreviations

CYP, cytochrome P450; PB, phenobarbital; PXR, pregnane X receptor; CAR, constitutive androstane receptor; CXR, chicken xenobiotic receptor; PBRU, phenobarbital-responsive enhancer unit; ALAS-1, δ-aminolevulinate synthase; AF-1/2, activation function-1/2; LXR, liver X receptor; PB, phenobarbital; XRS, xenochemical response signal; GFP, green fluorescent protein; PPAR, peroxisome-proliferator activated receptor; FXR, farnesoid X receptor.

## Competing interests

The authors declare that they have no competing interests.

## Authors' contributions

CH carried out the cellular localization assays, cloned the various CXR mutants, performed the reporter gene and the electrophoretic-mobility shift assays as well as the transcriptional regulation studies. SB did the various screens for further chicken xenobiotic-sensing nuclear receptors. AR performed the RNAi experiments. RL and MO isolated the CXR antibody and carried out the protein stabilization and localization assays. MRK helped with the RNAi experiments. MP and CG helped with the CV-1 cell transactivation assays. UAM conceived of the study, and participated in its design and coordination. All authors read and approved the final manuscript.
